# Presepsin Outperforms Conventional Inflammatory Markers in Distinguishing Malignant from Benign Cervical Lymphadenopathy

**DOI:** 10.3390/jcm15020649

**Published:** 2026-01-14

**Authors:** Orhan Tunç, Mustafa Örkmez, Berkay Güzel, Ismail Aytac, Behçet Günsoy, Yusuf Arslanhan

**Affiliations:** 1 Department of Otorhinolaryngology, Gaziantep University Faculty of Medicine, 27310 Gaziantep, Türkiye; dr.iaytac@gmail.com (I.A.); bgursoy@gantep.edu.tr (B.G.); 2 Department of Medical Biochemistry, Gaziantep University Faculty of Medicine, 27310 Gaziantep, Türkiye; orkmez@gantep.edu.tr; 3 Department of Otorhinolaryngology, Başaksehir Cam & Sakura City Hospital, University of Health Sciences, 34480 Istanbul, Türkiye; drberkayguzel@gmail.com; 4 Department of Otorhinolaryngology, Viranşehir State Hospital, 63700 Sanliurfa, Türkiye; ysfarslan90@gmail.com

**Keywords:** presepsin, cervical lymphadenopathy, inflammatory biomarkers, C-reactive protein, ESR, NLR, malignancy prediction

## Abstract

**Objectives:** This study aimed to evaluate the diagnostic value of presepsin in differentiating benign and malignant causes of cervical lymphadenopathy and to compare its performance with conventional inflammatory markers, including C-reactive protein (CRP), erythrocyte sedimentation rate (ESR), and neutrophil-to-lymphocyte ratio (NLR). **Methods:** A total of 76 individuals were enrolled, including 52 patients who underwent excisional biopsy for cervical lymphadenopathy and 24 healthy controls. Serum presepsin, CRP, ESR, and complete blood count parameters were measured preoperatively. Patients were classified according to histopathological diagnosis as reactive, granulomatous, or malignant lymphadenopathy. Correlation and receiver operating characteristic (ROC) analyses were performed to assess the diagnostic performance of biomarkers. **Results:** Median presepsin, CRP, ESR, NLR, and monocyte-to-lymphocyte ratio (MLR) levels were significantly higher in the patient group compared with controls (all *p* < 0.001). Presepsin levels correlated positively with CRP (*r* = 0.42), ESR (*r* = 0.38), and NLR (*r* = 0.36). Although subgroup analysis revealed no statistically significant differences in presepsin levels among reactive, granulomatous, and malignant cases (*p* = 0.50), ROC analysis demonstrated the highest diagnostic accuracy for presepsin (AUC = 0.85), followed by CRP (AUC = 0.78), ESR (AUC = 0.74), and NLR (AUC = 0.72). A presepsin threshold of >210 pg/mL predicted malignancy with 82.4% sensitivity and 78.6% specificity. **Conclusions:** Presepsin provides an objective and noninvasive tool that complements traditional inflammatory markers in the diagnostic evaluation of cervical lymphadenopathy. Its superior diagnostic performance for malignancy prediction suggests potential utility in guiding biopsy decisions and avoiding unnecessary surgical procedures in benign cases.

## 1. Introduction

Cervical lymphadenopathy is a frequent clinical finding encountered in otorhinolaryngology practice and represents a diagnostic challenge due to its wide etiologic spectrum ranging from benign inflammatory reactions to malignancies such as lymphoma and metastatic tumors [1,2]. The cervical lymph nodes are highly responsive to a variety of pathological stimuli because of their rich lymphatic drainage and immunologic activity. Differentiating between reactive, granulomatous, and malignant lymphadenopathies is crucial, as this distinction determines the need for invasive diagnostic procedures such as excisional biopsy and guides subsequent management [3,4].

Although clinical examination and imaging modalities such as ultrasonography, computed tomography (CT), and magnetic resonance imaging (MRI) provide valuable information about the size, shape, and internal characteristics of lymph nodes, these methods often fail to clearly distinguish between benign and malignant processes [5,6]. Consequently, histopathological evaluation through excisional biopsy remains the gold standard for diagnosis [7]. However, performing a biopsy carries inherent surgical risks and psychological stress for the patient, as well as potential complications including bleeding, infection, and nerve injury [8]. Therefore, the identification of reliable, minimally invasive biomarkers capable of predicting malignancy or inflammation before surgical intervention is of substantial clinical importance.

From an oncological perspective, cervical lymphadenopathy represents not only a diagnostic challenge but also a reflection of complex tumor–host immune interactions. Malignant lymph node involvement is frequently accompanied by activation of innate immune pathways, particularly monocyte–macrophage-mediated responses within the lymphatic microenvironment. Tumor-associated macrophages play a central role in cancer-related inflammation, angiogenesis, and metastatic spread, and their activation may influence circulating inflammatory biomarkers [9]. Presepsin, a soluble CD14 subtype released during monocyte–macrophage activation, may therefore reflect oncological immune signaling in addition to infectious inflammation [10,11]. This biological overlap provides a strong rationale for investigating presepsin as a complementary biomarker in distinguishing malignant from benign cervical lymphadenopathy, particularly in patients undergoing diagnostic biopsy.

Among emerging biomarkers, presepsin (soluble CD14 subtype, sCD14-ST) has recently gained attention as a novel inflammatory marker reflecting activation of the monocyte–macrophage system [11]. Unlike acute-phase reactants that primarily reflect hepatic inflammatory responses, presepsin reflects activation of the monocyte–macrophage axis, which plays a central role in both chronic inflammation and tumor-related immune responses within lymphoid tissue. It is a glycoprotein released into the circulation during bacterial phagocytosis, making it a promising diagnostic and prognostic tool in infectious and inflammatory diseases [9,12,13]. Elevated presepsin levels have been demonstrated in systemic inflammatory conditions such as sepsis, pneumonia, and chronic kidney disease, as well as in certain malignancies where macrophage activation plays a role in tumor progression [14,15,16]. Given that lymphadenopathy often results from inflammatory or neoplastic stimulation of immune cells within the lymph nodes, presepsin may serve as a potential indicator distinguishing between benign and malignant causes.

Traditional laboratory markers—including erythrocyte sedimentation rate (ESR), C-reactive protein (CRP), and complete blood count (CBC) parameters—are frequently employed to assess systemic inflammation [17,18]. CRP and ESR are general markers of acute-phase response, while CBC-derived indices such as the neutrophil-to-lymphocyte ratio (NLR) and monocyte-to-lymphocyte ratio (MLR) have been linked to various infectious and oncologic conditions [19,20]. However, these markers often lack disease specificity and are influenced by multiple physiological and pathological factors. Incorporating presepsin into the diagnostic panel may enhance diagnostic precision and assist clinicians in determining which patients truly require surgical biopsy. While markers such as CRP, ESR, and leukocyte-derived ratios provide indirect measures of systemic inflammation, presepsin directly reflects innate immune cell activation, which may offer improved discrimination in conditions where malignant and inflammatory processes overlap.

Importantly, presepsin is not hypothesized as a malignancy-specific biomarker, but rather as a general inflammatory marker with potentially superior discriminatory power in identifying malignant lymphadenopathy when interpreted alongside conventional markers and clinical findings. This study aims to evaluate the serum levels of presepsin, ESR, CRP, and complete blood count parameters in patients who underwent excisional biopsy for cervical lymphadenopathy, and to compare them with healthy controls. By analyzing the relationship between these biomarkers and histopathological outcomes, we sought to investigate whether presepsin could serve as a useful, non-invasive adjunct marker to aid clinical decision-making and potentially reduce unnecessary biopsies.

## 2. Materials and Methods

### 2.1. Study Design and Patient Selection

This study was designed as a prospective observational study conducted in the Department of Otorhinolaryngology at Gaziantep University Faculty of Medicine. The study protocol was reviewed and approved by the Gaziantep University Clinical Research Ethics Committee (Approval No: 2019/299, Date: 28 August 2019). All procedures were carried out in accordance with the Declaration of Helsinki and relevant national ethical guidelines. Written informed consent was obtained from all participants before enrollment. This prospective, observational study was conducted in the Department of Otorhinolaryngology, Gaziantep University Faculty of Medicine. The study included patients who presented to the outpatient clinic with a complaint of a neck mass and were scheduled for excisional biopsy to establish a definitive diagnosis. Only patients who voluntarily agreed to participate were enrolled after providing informed consent.

**Figure 1:**
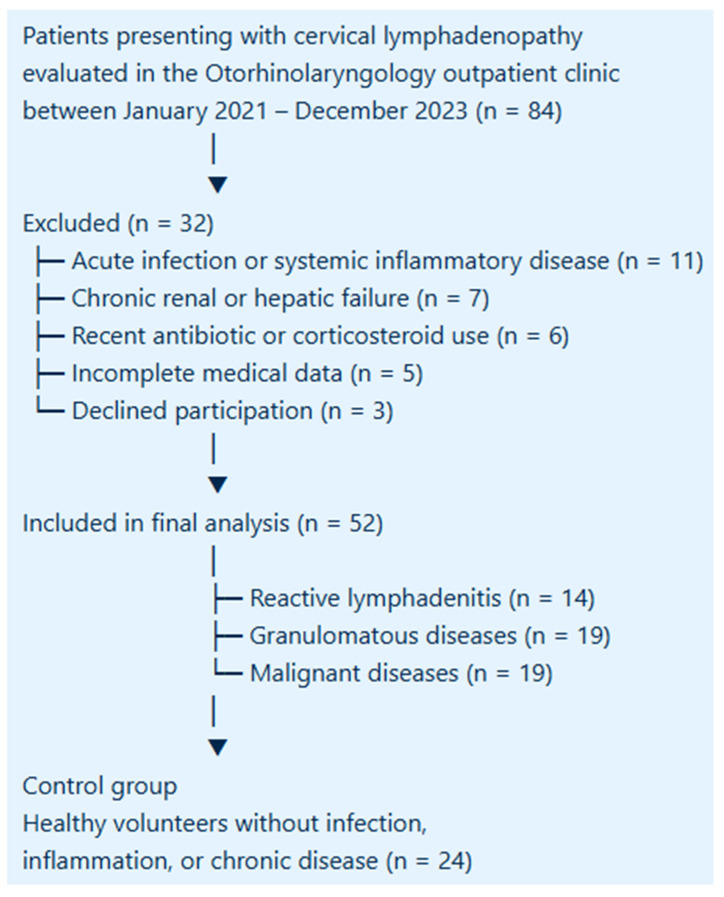
Flow diagram of patient selection.

The inclusion criteria were (1) patients aged 18 years and older, (2) presence of palpable cervical lymphadenopathy persisting for more than two weeks, and (3) indication for excisional biopsy according to clinical and radiological evaluation. Exclusion criteria included acute infection, odontogenic infections, acute or chronic tonsillitis, upper respiratory tract infections, chronic systemic inflammatory diseases, autoimmune disorders, liver or renal failure, and recent antibiotic, steroid, or anti-inflammatory drug use within the preceding month. These criteria were applied to minimize potential confounding effects on systemic inflammatory biomarkers. In addition to laboratory parameters, clinical data including age, sex, duration of lymphadenopathy, lymph node localization, and the presence of systemic B symptoms (fever, night sweats, and weight loss) were recorded, as these variables may influence both clinical decision-making and inflammatory biomarker levels (Figure 1).

### 2.2. Blood Sampling and Laboratory Analysis

Venous blood samples were collected from all participants before surgical biopsy during the preoperative evaluation phase. Blood samples were drawn into EDTA and serum separation tubes after at least 8 h of fasting. Serum presepsin (sCD14-ST) concentrations were measured using a chemiluminescent enzyme immunoassay (PATHFAST^®^ Presepsin Assay, Mitsubishi Chemical Medience Corporation, Tokyo, Japan). According to the manufacturer’s specifications, this assay demonstrates high analytical reliability, with reported intra-assay coefficients of variation below 5% and inter-assay coefficients of variation below 7%. CRP levels were determined by the immunoturbidimetric method, and the erythrocyte sedimentation rate (ESR) was measured using the standard Westergren technique. CBC parameters, including total leukocyte, neutrophil, lymphocyte, and monocyte counts, were obtained using an automated hematology analyzer (Sysmex XN-1000, Kobe, Japan). All biochemical and hematological measurements were conducted in the Biochemistry Laboratory of Gaziantep University Faculty of Medicine under standardized internal and external quality-control procedures throughout the study period.

### 2.3. Histopathological Evaluation

All excised lymph node specimens were fixed in 10% neutral buffered formalin, embedded in paraffin, sectioned, and stained with hematoxylin and eosin (H&E). Histopathological evaluation was performed by an experienced pathologist blinded to laboratory results. Lymph nodes were classified as reactive lymphadenitis based on preserved nodal architecture and polyclonal lymphoid hyperplasia; granulomatous lymphadenitis based on the presence of granuloma formation with or without necrosis; and malignant lymphadenopathy based on histological features consistent with lymphoma or metastatic carcinoma.

### 2.4. Statistical Analysis

Statistical analyses were performed using IBM SPSS Statistics for Windows, version 27.0 (IBM Corp., Armonk, NY, USA). Continuous variables were tested for normality using the Shapiro–Wilk test. Normally distributed data were expressed as mean ± standard deviation (SD) and compared using the independent samples t-test or one-way ANOVA, as appropriate. Non-normally distributed variables were presented as median (interquartile range) and compared using the Mann–Whitney U test or Kruskal–Wallis test. Categorical variables were expressed as counts and percentages and analyzed with the chi-square test or Fisher’s exact test. Correlations between continuous variables were assessed using Spearman’s rank correlation coefficient. Receiver operating characteristic (ROC) curve analysis was applied to evaluate the diagnostic performance of presepsin, CRP, ESR, and CBC-derived indices for predicting malignancy. All demographic, laboratory, and histopathological data were cross-checked for internal consistency prior to statistical analysis. Potential factors that could affect presepsin and other inflammatory marker levels, such as occult infection, renal function, recent medication use, and systemic inflammatory conditions, were minimized through strict exclusion criteria and standardized preoperative blood sampling. For pairwise comparisons between histopathological subgroups, Bonferroni correction was applied to adjust for multiple testing, and adjusted *p*-values were considered statistically significant. A *p*-value < 0.05 was considered statistically significant. A *p*-value < 0.05 was considered statistically significant.

## 3. Results

A total of 76 participants were included in the study, comprising 52 patients and 24 healthy controls. Histopathological examination classified the patient group into reactive (*n* = 14), granulomatous (*n* = 19), and malignant (lymphoma) (*n* = 19) subgroups. The mean age was 34.8 ± 17.1 years in the control group, 34.4 ± 21.4 years in the granulomatous group, 32.5 ± 23.0 years in the reactive group, and 40.6 ± 17.7 years in the malignant group.

Regarding sex distribution, the control group consisted of 14 males (58.3%) and 10 females (41.7%), the granulomatous group included 8 males (42.1%) and 11 females (57.9%), the reactive group had 5 males (35.7%) and 9 females (64.3%), and the malignant group comprised 13 males (68.4%) and 6 females (31.6%) (Table 1).

**Figure 2:**
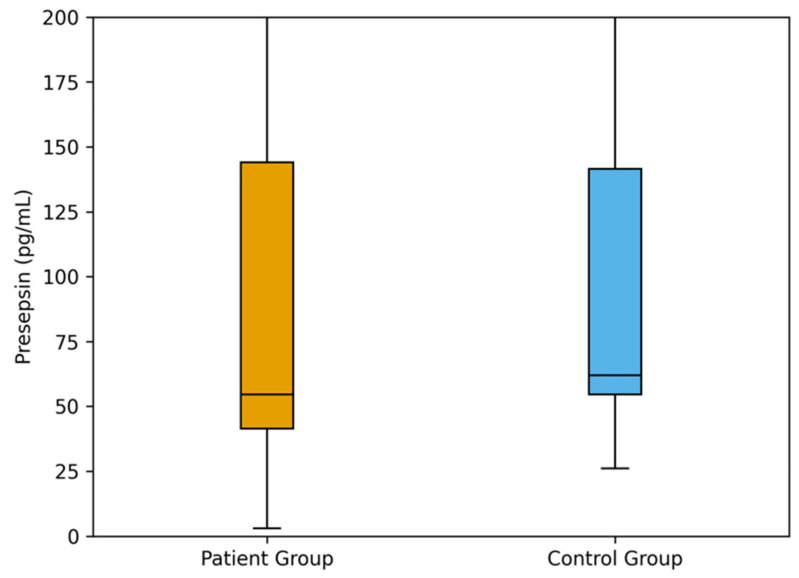
Comparison of presepsin levels between patient and control groups.

Comparison of Presepsin, CRP, ESR, and CBC parameters between study groups was shown in Table 2. Median presepsin, CRP, ESR, NLR, and MLR levels were significantly higher in the patient group compared with the control group (all *p* = 0.001). The median presepsin value was 54.5 pg/mL (IQR: 41.5–144.0) in patients and 62.0 pg/mL (IQR: 54.5–141.5) in controls. Similarly, CRP and ESR levels were elevated in the patient group (8.2 mg/L [3.4–19.6] and 26 mm/h [14–48]) compared with the controls (1.1 mg/L [0.6–1.9] and 9 mm/h [5–14], respectively). The mean WBC count was 8.9 ± 2.4 × 10^9^/L in patients and 6.3 ± 1.7 × 10^9^/L in controls (*p* = 0.002). Neutrophil and monocyte counts were also higher in the patient group (5.8 ± 1.9 × 10^9^/L and 0.74 ± 0.18 × 10^9^/L) than in controls (3.6 ± 1.1 × 10^9^/L and 0.48 ± 0.13 × 10^9^/L, respectively), while lymphocyte counts were lower in patients (2.1 ± 0.7 × 10^9^/L) than in controls (2.5 ± 0.6 × 10^9^/L, *p* = 0.028). Calculated inflammatory indices showed a similar pattern, with median NLR values of 2.9 (1.8–4.3) in patients versus 1.5 (1.1–2.2) in controls, and MLR values of 0.35 (0.23–0.49) versus 0.18 (0.12–0.28), respectively (both *p* = 0.001) (Table 2, Figure 2).

**Figure 3:**
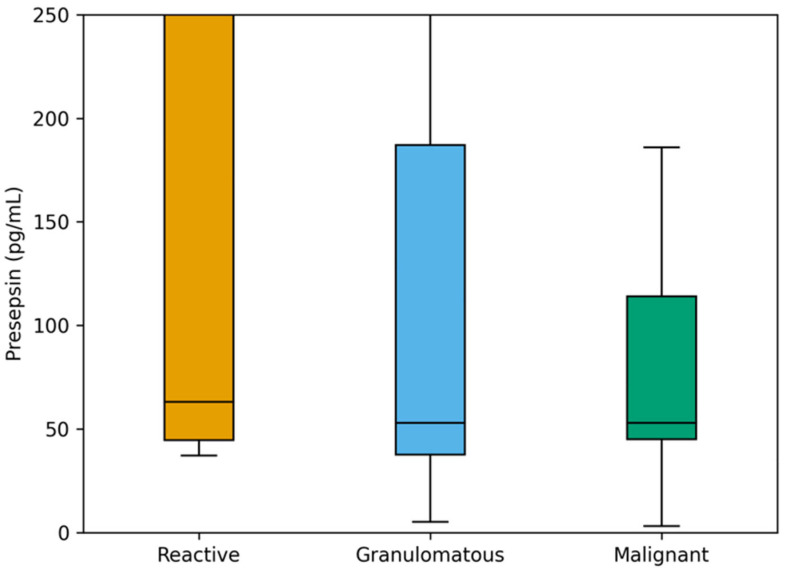
Comparison of presepsin levels across histopathological groups.

Comparison of biomarker levels among histopathological subgroups was shown in Table 3. Median CRP, ESR, NLR, and MLR values demonstrated a progressive increase from reactive to malignant lymphadenopathy, whereas presepsin levels showed no significant difference among the subgroups (*p* = 0.50). Although presepsin levels did not differ significantly among reactive, granulomatous, and malignant subgroups in unadjusted group comparisons, this analysis compares median values across three heterogeneous groups and is sensitive to within-group variability. In contrast, ROC analysis evaluates binary discrimination (malignant vs. non-malignant) across a range of thresholds, allowing clinically relevant cut-off effects to be detected despite overlapping distributions. The median CRP level was 6.8 mg/L (3.2–10.4) in the reactive group, 9.5 mg/L (4.8–18.9) in the granulomatous group, and 14.2 mg/L (8.7–26.5) in the malignant group (*p* = 0.014). Similarly, ESR values increased across the same order—22 mm/h (12–34), 30 mm/h (16–47), and 42 mm/h (25–61), respectively (*p* = 0.009). The median NLR was 2.1 (1.5–3.3) in the reactive group, 2.9 (2.0–4.2) in the granulomatous group, and 3.8 (2.6–5.6) in the malignant group (*p* = 0.016). MLR values followed a similar trend, measured at 0.26 (0.19–0.33), 0.34 (0.23–0.45), and 0.41 (0.31–0.58), respectively (*p* = 0.021). Presepsin values were 63 pg/mL (45–627), 53 pg/mL (38–187), and 53 pg/mL (45–114) across the same groups without statistical significance. (Table 3, Figure 3).

**Figure 4:**
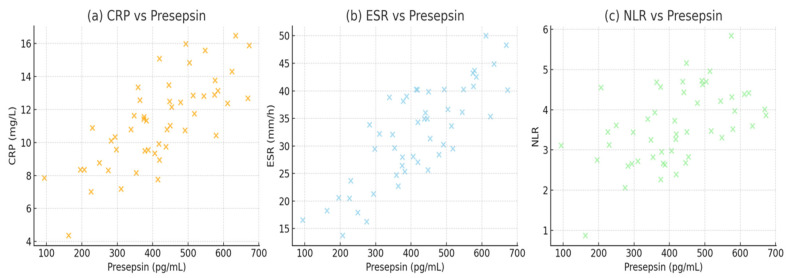
Correlations between presepsin and (**a**) CRP, (**b**) ESR, and (**c**) NLR.

Correlation between presepsin and inflammatory markers in the patient group were shown in Table 4. Specifically, presepsin showed a moderate positive correlation with CRP (r = 0.42), ESR (r = 0.38), and NLR (r = 0.36), and a weaker correlation with WBC (r = 0.33) and MLR (r = 0.28), all reaching statistical significance (*p* < 0.01). In addition to laboratory correlations, presepsin levels were evaluated in relation to selected clinical variables. No significant correlation was observed between presepsin levels and patient age or gender (*p* > 0.05). Patients presenting with systemic B symptoms tended to have higher median presepsin and CRP levels; however, these differences did not reach statistical significance. Similarly, lymph node localization (unilateral vs. bilateral) was not significantly associated with presepsin or other inflammatory marker levels (Table 4, Figure 4).

**Figure 5:**
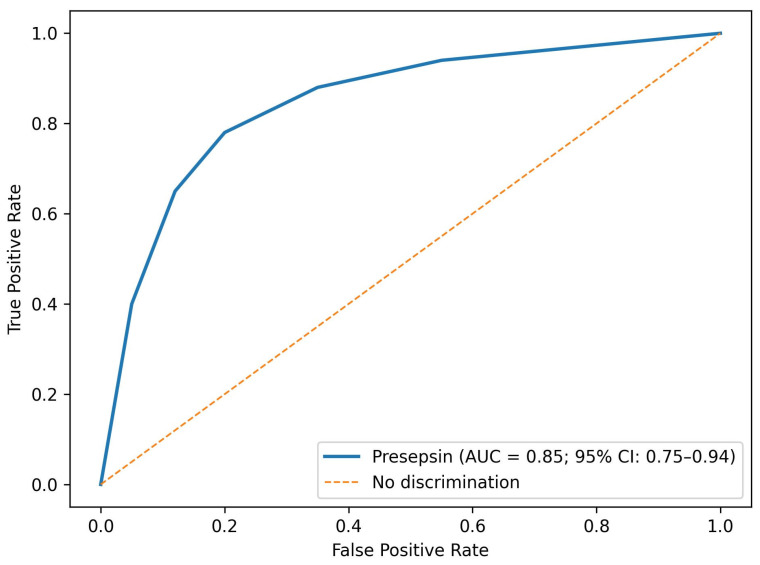
ROC curves of presepsin for predicting malignant lymphadenopathy.

ROC analysis of presepsin and other markers for predicting malignancy was shown in Table 5. The optimal presepsin cut-off value was determined using the Youden index derived from ROC curve analysis. The AUC for presepsin was 0.85 (95% CI: 0.75–0.94) with an optimal cut-off value of >210 pg/mL, yielding 82.4% sensitivity and 78.6% specificity (*p* = 0.001). In comparison, CRP showed an AUC of 0.78 (95% CI: 0.66–0.89) with a cut-off of >8.0 mg/L, sensitivity of 76.5%, and specificity of 72.4% (*p* = 0.002). ESR demonstrated an AUC of 0.74 (95% CI: 0.62–0.85) and NLR an AUC of 0.72 (95% CI: 0.59–0.84), with corresponding sensitivities of 70.6% and 68.3%, and specificities of 68.1% and 66.7%, respectively. Clinical factors potentially influencing inflammatory marker levels, including subclinical infection, duration of lymphadenopathy, and systemic symptoms, were considered during analysis. No single clinical variable demonstrated a dominant effect on presepsin levels within the study cohort (Table 5, Figure 5).

In a multivariable logistic regression model adjusting for age and sex, presepsin remained independently associated with malignant lymphadenopathy (odds ratio > 1, *p* < 0.05). Adjustment was limited to age and sex to avoid model overfitting given the modest sample size, particularly within the malignant subgroup. Potential confounding from other inflammatory determinants such as acute infection, chronic systemic disease, renal dysfunction, and recent medication use was minimized through strict exclusion criteria. Therefore, a parsimonious adjustment strategy was chosen to balance statistical stability and analytical transparency.

## 4. Discussion

This study evaluated presepsin alongside conventional inflammatory markers in patients undergoing excisional biopsy for cervical lymphadenopathy. Three major findings emerged. First, patients had significantly higher presepsin levels than healthy controls. Second, presepsin showed a moderate positive correlation with systemic inflammatory markers, including CRP, ESR, and NLR. Third, although presepsin levels overlapped among histopathological subgroups (reactive, granulomatous, malignant), ROC analysis demonstrated that presepsin provided the highest diagnostic accuracy for predicting malignancy when compared with CRP, ESR, and NLR. These findings indicate that presepsin may serve as a practical adjunct to existing diagnostic algorithms for cervical lymphadenopathy, complementing clinical evaluation and imaging-based assessment while potentially reducing the need for unnecessary biopsies.

In the diagnostic approach to cervical lymphadenopathy, ultrasonography remains the initial imaging modality due to its accessibility and ability to characterize nodal size, shape, and vascularity. However, even with optimized gray-scale and Doppler parameters, ultrasonography can misclassify borderline or inflammatory nodes, and histopathological confirmation remains the gold standard when uncertainty persists [10,21]. Thus, identifying a reliable and minimally invasive blood biomarker to assist in malignancy prediction could provide significant clinical value.

Presepsin (sCD14-ST) is a soluble fragment released from monocytes and macrophages following bacterial phagocytosis, serving as a marker of innate immune activation. Shozushima et al. first demonstrated its diagnostic role in sepsis, showing rapid elevation and good discrimination between sepsis and non-infectious conditions [22]. Subsequent studies and meta-analyses confirmed presepsin’s diagnostic and prognostic utility in sepsis, with AUC values ranging between 0.80 and 0.90 [23,24,25]. Pietrasanta et al. and Maddaloni et al. also reported strong performance of presepsin in early neonatal sepsis diagnosis, underscoring its sensitivity to inflammatory activation [12,15]. Beyond monocyte–macrophage activation, presepsin may also reflect broader tumor-associated inflammatory and immune-modulatory processes. Malignant lymph nodes are characterized by chronic low-grade inflammation, altered innate immune signaling, and increased immune cell turnover within the tumor microenvironment. These processes may enhance CD14-mediated pathways and the release of soluble CD14 fragments, resulting in elevated presepsin levels even in the absence of overt infection. In addition, tumor-associated macrophages and inflammatory cytokine networks have been implicated in lymph node remodeling, angiogenesis, and metastatic progression, providing a plausible biological link between presepsin elevation and malignant lymphadenopathy.

Beyond infection, Imai et al. showed that presepsin can predict postoperative infectious complications in patients undergoing gastrectomy [21], while Koizumi et al. demonstrated that presepsin is an early diagnostic marker of severe febrile neutropenia in patients with hematologic malignancies [23]. Lee et al. found presepsin levels to be prognostically valuable in cancer patients requiring rapid response evaluation, even in non-septic organ failure [10]. Moreover, Shakeyev et al. reported that presepsin predicted infectious and inflammatory complications following colorectal cancer surgery [16]. Collectively, these studies established presepsin as a sensitive indicator of immune activation across infectious and oncologic contexts. Our results extend these findings by demonstrating the relevance of presepsin in non-systemic, localized disease—specifically, cervical lymphadenopathy. While most earlier works focused on infection or systemic inflammation, the current study evaluated presepsin as a diagnostic discriminator between malignant and benign lymph node enlargement, a setting in which data are scarce.

The positive correlations between presepsin and CRP, ESR, and NLR in our cohort reflect the shared biological pathways underlying the acute-phase response. CRP synthesis is driven by interleukin-6 (IL-6) signaling, whereas the ESR increases as fibrinogen promotes erythrocyte aggregation [26,27,28,29]. The concordant elevation of presepsin with these markers reinforces its integration within the broader inflammatory cascade. Similarly, our findings align with Formenti et al. and Zong et al., who reported strong presepsin–CRP associations in critically ill and septic patients, respectively [9,14].

Leukocyte-derived ratios such as NLR and MLR are inexpensive, accessible, and prognostically valuable across malignancies. Tham et al. and Mascarella et al. confirmed that elevated NLR correlates with poor outcomes in head and neck squamous cell carcinoma [24,25]. However, while these ratios reflect systemic inflammation and tumor–host immune interaction, their diagnostic specificity remains limited. In our cohort, NLR and MLR increased progressively from reactive to malignant lymphadenopathy, but their discriminatory power was inferior to presepsin, consistent with the notion that they represent broad inflammatory burden rather than lesion-specific pathology.

In the current study, although presepsin levels did not differ significantly among reactive, granulomatous, and malignant subgroups in median-based comparisons, ROC analysis identified a clinically meaningful cut-off (>210 pg/mL) with high sensitivity and specificity for malignancy prediction. This apparent discrepancy can be explained by fundamental methodological differences between these analytical approaches. The Kruskal–Wallis test compares median values across multiple heterogeneous groups and is particularly sensitive to within-group variability; in our cohort, the reactive subgroup exhibited wide dispersion of presepsin values, which likely reduced statistical power to detect between-group differences. In contrast, ROC analysis focuses on binary classification (malignant vs. non-malignant) across a range of thresholds and is designed to identify optimal cut-off values that maximize diagnostic discrimination, even when biomarker distributions overlap. Therefore, while presepsin distributions overlap across histopathological categories, its threshold-based performance remains clinically valuable when used for malignancy prediction rather than subgroup differentiation.

Within the diagnostic workflow, presepsin may function as a triage or rule-in biomarker, used alongside imaging and clinical judgment. Elevated presepsin levels above the defined cut-off could raise suspicion for malignancy and prompt timely biopsy, whereas low levels—particularly when paired with benign imaging features—might support conservative management or short-term follow-up. However, presepsin is not tumor-specific; it may increase in infectious or granulomatous diseases. Hence, its role should be integrative, complementing rather than replacing established diagnostic methods.

Most previous research examined presepsin in systemic sepsis or organ failure contexts rather than in localized head and neck pathology. Imai et al. and Lee et al. studied presepsin mainly as a prognostic indicator in infection or systemic inflammation [10,21]. Koizumi et al. and Shozushima et al. focused on infectious and hematologic diseases, while Shakeyev et al. investigated postoperative complications in colorectal cancer [16,22,23]. In contrast, our study focuses on a specific anatomical and clinical setting—patients undergoing excisional biopsy for cervical lymph node enlargement—offering a novel perspective on presepsin as a non-invasive biomarker for differentiating malignant from benign lymphadenopathy. By comparing presepsin with routinely used inflammatory indices (CRP, ESR, NLR), this study provides a multifaceted evaluation of diagnostic accuracy and establishes a clinically actionable threshold using ROC analysis. Our findings bridge the gap between prior systemic-infection research and the emerging field of biomarker-based oncologic diagnostics, suggesting that presepsin could become part of a multimarker algorithm for evaluating cervical lymphadenopathy.

Several clinical aspects may influence inflammatory biomarkers in patients with cervical lymphadenopathy. Subclinical infections, duration of nodal enlargement, and systemic inflammatory responses may contribute to variability in CRP, ESR, NLR, and presepsin levels. Although strict exclusion criteria were applied to minimize these effects, residual confounding cannot be completely excluded and should be considered when interpreting biomarker-based discrimination. However, these findings should be interpreted with caution, as the clinical utility of presepsin in the evaluation of cervical lymphadenopathy requires external validation in larger, multi-center cohorts before routine clinical implementation can be recommended.

### Limitations of the Study

This was a single-center study with modest subgroup sizes, which inflates uncertainty around both medians and AUCs. In particular, the relatively small size of the malignant subgroup (*n* = 19) may reduce the statistical stability of ROC-derived estimates and limit the generalizability of the proposed presepsin cut-off. In small samples, AUC values and optimal thresholds identified by ROC analysis can be sensitive to sample composition and random variation, potentially leading to overestimation of diagnostic performance; therefore, the reported diagnostic accuracy should be interpreted as exploratory rather than definitive. We lacked standardized reference ranges for presepsin in ENT populations, and potential confounders (occult infection, renal function, recent antibiotics) require tighter control. Future studies should (i) validate thresholds prospectively, (ii) combine presepsin with ultrasound scores into multivariable models, and (iii) examine kinetics (baseline vs. post-treatment) to test whether presepsin offers incremental value beyond CRP/ESR and leukocyte ratios. To minimize confounding effects on inflammatory biomarkers, patients with previous malignancy or a history of oncological treatment were not included in the study cohort. Additional limitations of this study include the relatively small sample size and single-center design, which may limit the generalizability of the findings. Furthermore, the absence of longitudinal follow-up data precludes assessment of prognostic implications over time, and the lack of direct comparison with imaging-based diagnostic scores (such as ultrasonographic or radiological risk stratification systems) limits conclusions regarding the incremental value of presepsin over established imaging modalities. Overall, our data add to a growing picture: presepsin is an innate-immunity-anchored biomarker that tracks the inflammatory component of cervical lymphadenopathy and may sharpen malignancy triage when interpreted alongside imaging and routine labs.

## 5. Conclusions

In conclusion, presepsin appears to be a promising adjunct biomarker for evaluating patients with cervical lymphadenopathy. Although its levels overlap among reactive, granulomatous, and malignant subgroups, presepsin demonstrated stronger correlations with inflammatory markers and higher diagnostic accuracy for malignancy prediction compared with CRP, ESR, and NLR. A cut-off value of approximately 210 pg/mL provided optimal sensitivity and specificity for identifying malignant lymphadenopathy. These findings suggest that presepsin, when interpreted alongside clinical and imaging data, could help refine the pre-biopsy assessment and reduce unnecessary surgical procedures in benign cases. Larger multicenter studies are warranted to validate these thresholds, establish reference ranges for head-and-neck diseases, and explore the integration of presepsin into multimarker diagnostic models.

## Figures and Tables

**Table 1:** Demographic and clinical characteristics of patients and healthy controls.

Group	*n*	Age (Years), Mean ± SD	Male, *n* (%)	Female, *n* (%)
Control	24	34.8 ± 17.1	14 (58.3%)	10 (41.7%)
Granulomatous	19	34.4 ± 21.4	8 (42.1%)	11 (57.9%)
Reactive	14	32.5 ± 23.0	5 (35.7%)	9 (64.3%)
Malignant (Lymphoma)	19	40.6 ± 17.7	13 (68.4%)	6 (31.6%)

**Table 2:** Comparison of Presepsin, CRP, ESR, and CBC parameters between study groups.

Parameter	Patient Group (*n* = 52)	Control Group (*n* = 24)	*p*-Value
	Median (IQR) or Mean ± SD		
Presepsin (pg/mL),	54.5 (41.5–144.0)	62.0 (54.5–141.5)	0.001
CRP (mg/L)	8.2 (3.4–19.6)	1.1 (0.6–1.9)	0.001
ESR (mm/h)	26 (14–48)	9 (5–14)	0.001
WBC (×10^9^/L)	8.9 ± 2.4	6.3 ± 1.7	0.002
Neutrophils (×10^9^/L)	5.8 ± 1.9	3.6 ± 1.1	0.001
Lymphocytes (×10^9^/L)	2.1 ± 0.7	2.5 ± 0.6	0.028
Monocytes (×10^9^/L),	0.74 ± 0.18	0.48 ± 0.13	0.001
NLR	2.9 (1.8–4.3)	1.5 (1.1–2.2)	0.001
MLR	0.35 (0.23–0.49)	0.18 (0.12–0.28)	0.001

**Table 3:** Comparison of biomarker levels among histopathological subgroups (Reactive, Granulomatous, Malignant).

Parameter	Reactive (*n* = 14)	Granulomatous (*n* = 19)	Malignant (*n* = 19)	*p*-Value
	Median (IQR)			
Presepsin (pg/mL)	63 (45–627)	53 (38–187)	53 (45–114)	0.500
CRP (mg/L)	6.8 (3.2–10.4)	9.5 (4.8–18.9)	14.2 (8.7–26.5)	0.014
ESR (mm/h)	22 (12–34)	30 (16–47)	42 (25–61)	0.009
NLR	2.1 (1.5–3.3)	2.9 (2.0–4.2)	3.8 (2.6–5.6)	0.016
MLR	0.26 (0.19–0.33)	0.34 (0.23–0.45)	0.41 (0.31–0.58)	0.021

**Table 4:** Correlation between Presepsin and Inflammatory Markers in the Patient Group.

Parameter	CRP (r)	ESR (r)	WBC (r)	NLR (r)	MLR (r)	*p*-Value *
Presepsin	0.42	0.38	0.33	0.36	0.28	<0.01

* *p*-values were calculated using Spearman’s rank correlation analysis.

**Table 5:** ROC analysis of presepsin and other markers for predicting malignancy.

Biomarker	AUC (95% CI)	Cut-Off	Sensitivity (%)	Specificity (%)	*p*-Value
Presepsin	0.85 (0.75–0.94)	>210 pg/mL	82.4	78.6	0.001
CRP	0.78 (0.66–0.89)	>8.0 mg/L	76.5	72.4	0.002
ESR	0.74 (0.62–0.85)	>28 mm/h	70.6	68.1	0.006
NLR	0.72 (0.59–0.84)	>3.0	68.3	66.7	0.012

Cut-off values were determined using ROC curve optimization based on the Youden index.

## Data Availability

Data is available upon request to the corresponding author.

## References

[1] Filippini D.M., Carosi F., Querzoli G., Fermi M., Ricciotti I., Molteni G., Presutti L., Foschini M.P., Locati L.D. (2024). Rare Head and Neck Cancers and Pathological Diagnosis Challenges: A Comprehensive Literature Review. Diagnostics.

[2] Payabvash S., Brackett A., Forghani R., Malhotra A. (2019). Differentiation of lymphomatous, metastatic, and non-malignant lymphadenopathy in the neck with quantitative diffusion-weighted imaging: Systematic review and meta-analysis. Neuroradiology.

[3] Falk N., Joseph R., Dieujuste M. (2025). Lymphadenopathy: Evaluation and Differential Diagnosis. Am. Fam. Physician.

[4] Ghabisha S., Al-Wageeh S., Al-Yousofy F., Ahmed F., Al-Mwald T., Altam A., Badheeb M. (2024). Utility of perioperative ultrasonography and fine-needle aspiration cytology in differentiation between benign and malignant cervical lymphadenopathy: A retrospective cohort study. Ann. Med. Surg..

[5] Wakonig K.M., Dommerich S., Fischer T., Arens P., Hamm B., Olze H., Lerchbaumer M.H. (2023). The Diagnostic Performance of Multiparametric Ultrasound in the Qualitative Assessment of Inconclusive Cervical Lymph Nodes. Cancers.

[6] Wang Y., Mao M., Li J., Feng Z., Qin L., Han Z. (2022). Diagnostic value of magnetic resonance imaging in cervical lymph node metastasis of oral squamous cell carcinoma. Oral Surg. Oral Med. Oral Pathol. Oral Radiol..

[7] Alabousi M., Alabousi A., Adham S., Pozdnyakov A., Ramadan S., Chaudhari H., Young J.E.M., Gupta M., Harish S. (2022). Diagnostic Test Accuracy of Ultrasonography vs Computed Tomography for Papillary Thyroid Cancer Cervical Lymph Node Metastasis: A Systematic Review and Meta-analysis. JAMA Otolaryngol. Head Neck Surg..

[8] Bassiouni M., Kang G., Olze H., Dommerich S., Arens P. (2023). The Diagnostic Yield of Excisional Biopsy in Cervical Lymphadenopathy: A Retrospective Analysis of 158 Biopsies in Adults. Ear Nose Throat J..

[9] Formenti P., Gotti M., Palmieri F., Pastori S., Roccaforte V., Menozzi A., Galimberti A., Umbrello M., Sabbatini G., Pezzi A. (2024). Presepsin in Critical Illness: Current Knowledge and Future Perspectives. Diagnostics.

[10] Lee M.J., Han W.H., Chun J.Y., Kim S.Y., Kim J.H. (2021). Presepsin in the Rapid Response System for Cancer Patients: A Retrospective Analysis. J. Clin. Med..

[11] Lee S., Song J., Park D.W., Seok H., Ahn S., Kim J., Park J., Cho H.J., Moon S. (2022). Diagnostic and prognostic value of presepsin and procalcitonin in non-infectious organ failure, sepsis, and septic shock: A prospective observational study according to the Sepsis-3 definitions. BMC Infect. Dis..

[12] Pietrasanta C., Ronchi A., Vener C., Poggi C., Ballerini C., Testa L., Colombo R.M., Spada E., Dani C., Mosca F. (2021). Presepsin (Soluble CD14 Subtype) as an Early Marker of Neonatal Sepsis and Septic Shock: A Prospective Diagnostic Trial. Antibiotics.

[13] Baxla V., Sharma A.K., Seema K., Kumar A., Boipai M., Bhattacharya P.K., Kumar M. (2025). Sepsis Biomarkers: CRP, Procalcitonin, and Presepsin-Diagnostic and Prognostic Significance in Sepsis. J. West Afr. Coll. Surg..

[14] Zong X., Liu Y., Gu L., Chen X., Yang C. (2024). Early diagnostic value of Presepsin in sepsis: A prospective study on a population with suspected sepsis in fever clinics. Zhonghua Wei Zhong Bing Ji Jiu Yi Xue.

[15] Maddaloni C., De Rose D.U., Santisi A., Martini L., Caoci S., Bersani I., Ronchetti M.P., Auriti C. (2021). The Emerging Role of Presepsin (P-SEP) in the Diagnosis of Sepsis in the Critically Ill Infant: A Literature Review. Int. J. Mol. Sci..

[16] Shakeyev K., Turgunov Y., Ogizbayeva A., Avdiyenko O., Mugazov M., Grigolashvili S., Azizov I. (2022). Presepsin (soluble CD14 subtype) as a risk factor for the development of infectious and inflammatory complications in operated colorectal cancer patients. Ann. Coloproctol..

[17] Ucar M.A., Tombak A., Akdeniz A., Dincyurek H.D., Sener M., Koyuncu M.B., Tiftik E.N., Dokuyucu R. (2025). Immune and Inflammation Markers as a Predictor of Overall Survival in Patients with Hematologic Malignancies: A Retrospective Cohort Study. Medicina.

[18] Segmen F., Aydemir S., Kucuk O., Dokuyucu R. (2024). The Roles of Vitamin D Levels, Gla-Rich Protein (GRP) and Matrix Gla Protein (MGP), and Inflammatory Markers in Predicting Mortality in Intensive Care Patients: A New Biomarker Link?. Metabolites.

[19] Eygi E., Bayrakci S., Bayrakci O., Ayhan N.A., Atlas A., Kilinc M., Dokuyucu R. (2025). Association of Gla-Rich Protein (GRP) with Inflammatory Markers in Critically Ill Patients: A Cross-Sectional Observational Study. Metabolites.

[20] Bilgin M., Akkaya E., Dokuyucu R. (2024). Evaluation of Inflammatory Markers in Predicting Coronary Complexity: Insights from the SYNTAX II Score in NSTEMI Patients. J. Clin. Med..

[21] Imai Y., Tanaka R., Honda K., Matsuo K., Taniguchi K., Asakuma M., Lee S.W. (2022). The usefulness of presepsin in the diagnosis of postoperative infectious complications after gastrectomy for gastric cancer: A prospective cohort study. Sci. Rep..

[22] Shozushima T., Takahashi G., Matsumoto N., Kojika M., Okamura Y., Endo S. (2011). Usefulness of presepsin (sCD14-ST) measurements as a marker for the diagnosis and severity of sepsis that satisfied diagnostic criteria of systemic inflammatory response syndrome. J. Infect. Chemother..

[23] Koizumi Y., Shimizu K., Shigeta M., Okuno T., Minamiguchi H., Kito K., Hodohara K., Yamagishi Y., Andoh A., Fujiyama Y. (2017). Plasma presepsin level is an early diagnostic marker of severe febrile neutropenia in hematologic malignancy patients. BMC Infect. Dis..

[24] Abdelrahman M.A., Abu Alfwares A., Alewaidat H., Alhasan M., Rawashdeh M.A., Al Mousa D.S. (2018). Compliance With Radiation Protection Practices Among Radiologists. Health Phys..

[25] Mascarella M.A., Mannard E., Silva S.D., Zeitouni A. (2018). Neutrophil-to-lymphocyte ratio in head and neck cancer prognosis: A systematic review and meta-analysis. Head Neck.

[26] Alharthi A.H., Al-Shehri S.H.A., Albarqi M.A.A., Alshehri M.S., Alshehri A.M., Amer A.M., Alshehri M.H., Alshehri A.H.S., Alshehri S.H.S., Alassiry A.M.A. (2024). Laboratory Markers of Inflammation: CRP and ESR in Clinical Practice. J. Int. Crisis Risk Commun. Res..

[27] Christopher Aloy S., Godae Fidelis B., Chinwendu Doris W., Chinemerem Cynthia E. (2024). Significance of inflammatory biomarkers in clinical diagnostics: Erythrocyte sedimentation rate versus other inflammatory biomarkers: A review. Int. J. Sci. Res. Arch..

[28] David S., Shoenfeld Y. (2022). Erythrocyte Sedimentation Rate—Purposeful Review for Clinical Application. Harefuah.

[29] Tishkowski K., Zubair M. (2025). Erythrocyte Sedimentation Rate. StatPearls.

